# Association between dietary patterns and hypertension among Han and multi-ethnic population in southwest China

**DOI:** 10.1186/s12889-018-6003-7

**Published:** 2018-09-10

**Authors:** Yuan Ruan, Yongshou Huang, Qiang Zhang, Shu Qin, Xiaoxia Du, Yongxin Sun

**Affiliations:** 1 Department of nutrition and food hygiene, Centre for Disease Control and Prevention (CDC), No.158 Dongsi Street, Kunming, 650022 Yunnan China; 2 People’s hospital of Diqing Tibetan Autonomous Prefecture, No. 19 Chicika Street, Shangri-la, Diqing, 674400 Yunnan China; 3 CDC of Diqing Tibetan Autonomous Prefecture, No. 88 Ren’an Road, Shangri-la, Diqing, 674400 Yunnan China

**Keywords:** Dietary pattern, Factor analysis, Multi-ethnic, Hypertension, China

## Abstract

**Background:**

Different dietary patterns and the risks of hypertension in various diet exposures among multi-ethnic population in southwest China remain extremely scarce. The aim of this study is to identify dietary patterns and explore the association between dietary patterns and the risk of hypertension among Han and multi-ethnic population in southwest China.

**Methods:**

A representative sample of 3591 participants of Han, and multi-ethnic population were recruited by stratified cluster sampling in Diqing of Yunnan Province, southwest China from September 2012 to January 2013. Participants who were under 18 years old or who could not clearly answer the questions and those who used the anti-hypertensive medication were excluded from this survey. All participants reported their dietary intakes using validated food frequency questionnaires (FFQ), and their blood pressures were measured by standardized procedures. Hypertension was defined as systolic blood pressure (SBP) ≥ 140 mmHg and/or diastolic blood pressure (DBP) ≥ 90 mmHg. Dietary patterns were identified by factor analysis with principal component. Logistic regression was used to explore the association between dietary patterns and hypertension.

**Results:**

The overall prevalence of hypertension was 30.5% among Han and multi-ethnic population in Diqing, Yunnan Province. Three dietary patterns were identified in this study, defined as ‘Grassland healthy’, ‘Tuber and meat’, and ‘Fruit and vegetable’. Participants in the 5th quintile of the three dietary patterns were at a lower risk of hypertension compared with those in the 1st quintile. The odds ratio (OR) for the 5th quintile of ‘Grassland healthy’ pattern, ‘Tuber and meat’ and ‘Fruit and vegetable’ was 0.693 (95% CI: 0.537–0.893, *p* = 0.005), 0.678 (95% CI: 0.530–0.868, *p* = 0.002), 0.759 (95% CI: 0.593–0.970, *p* = 0.028), respectively. After further adjustment of participants’ age, the negative association between the ‘Grassland healthy’ pattern and the prevalence of hypertension persisted (OR = 0.703, 95% CI: 0.535–0.924, *p* = 0.012). However, the significant associations between the other two dietary patterns and hypertension disappeared.

**Conclusions:**

The ‘Grassland healthy’ dietary pattern is associated with lower risk of hypertension, whereas there is no significant associations between the other two dietary patterns and hypertension among Han and multi-ethnic population in Diqing of Yunnan province, southwest China.

**Electronic supplementary material:**

The online version of this article (10.1186/s12889-018-6003-7) contains supplementary material, which is available to authorized users.

## Background

Hypertension is a worldwide public health concern due to its high prevalence and risk for cardiovascular disease mortality both in developed and developing countries [1, 2]. There is 25% of the world’s adult population with hypertension and this figure is likely to increase to 29% by 2025. It is estimated that by 2025, the number of individuals with hypertension will be approximately 1.17 billion in developing countries, accounting for three-fourths of the world’s hypertensive population [3]. China is one of the world’s largest developing counties, with a population of 1.37 billion. According to a nationwide data from 2003 to 2012, the overall prevalence of hypertension was 26.7%, with the highest in east region (32.6%) followed by northeast region (31.8%) [4]. It is well-known that hypertension is a complex interaction of multiple genetic, environmental and behavior factors [5, 6]. Moreover, accumulating evidence suggested that unhealthy diet and lifestyle could play a significant role in the ongoing epidemics of obesity, hypertension and other symptoms of metabolic syndrome [7].

Although the role of diet in the development of hypertension was widely investigated, the majority of researches focus on the intake of individual foods and nutrients. Dietary pattern consisting of a number of food items is widely used as an alternative method to reflect habitual exposure of foods and nutrients as a whole to assess diet and disease relations [8]. Previous study suggest that the vegetarian dietary pattern which comprises foods rich in vegetables, fruits, grains, poultry, legumes, nuts, vegetable oils, soya, and possibly dairy products and/or eggs has been associated with a lower risk for developing hypertension [9]. The western dietary pattern rich in red meat and fats, sugary drinks, fast and processed food is related to a higher risk of hypertension [10]. In China, the traditional dietary greatly changed from low in fat and energy density to high in fat and energy density with the rapid growth of economy [11]. This remarkable nutrition transition may be a better explanation of the high rates of diet related non-communicable disease in the past decades in China [12]. Other studies have already analyzed the relationship between dietary patterns and the prevalence of hypertension, obesity, body weight and the diabetes in China [10, 13–16]. However, dietary patterns may vary between different cultural environments because of the differences in types of food consumed [17]. Therefore, more studies are needed to get further understandings of the association between dietary patterns and hypertension in China, especially in various diet exposures among multi-ethnic population in different places of China.

Yunnan is a relatively undeveloped province in southwest China. The prevalence of hypertension and years of life lost because of hypertension is 24.8% and 1.5 years, respectively, and hypertension also inflicts a considerable economic burden upon individual households and society in Yunnan [18]. However, issues on dietary and hypertension among Tibetan, Naxi and Lisu multi-ethnic population can’t be found. In this study, the dietary pattern and the associations between dietary pattern and hypertension are identified in order to give suggestions for prevention and control of hypertension in this area.

## Methods

### Study population, setting, and design

This study was a cross-sectional survey with the goal of assessing the association between dietary factors and hypertension among Han and multi-ethnic population in Diqing, which is in the northwest part in Yunnan Province, southwest China. The land area of Diqing is 23,870 km^2^, with a population of 405,000. It is the only Tibetan autonomous prefecture in Yunnan Province and the proportion of multi-ethnic population such as Tibetan, Lisu, Naxi is estimated as high as 88.5%. Participants were recruited with a stratified cluster sampling method in Diqing in Yunnan Province, southwest China from September in 2012 to January in 2013. All the three counties in Diqing were covered. First, each county was classified into urban area and rural area, and one street was randomly selected from urban areas. In rural area, towns were divided into three strata in terms of household income per capita, and one town was randomly selected from each strata. Then, two communities and two villages were randomly selected from each street or town. In each community or village, 97 households using cluster sampling were involved in this survey, and one member in the household was invited to take part in this survey with informed consent (Additional file 1: Informed consent). To be eligible, an individual had to be living in Diqing at the time of study and for at least 6 months of the previous year, be aged 18 years old or more, and be able to answer the questions without assistance (i.e. no major speech or neurological impairment). If the community or village failed to meet the sample size, they were merged by the nearby community or village to continue to finish this survey. Household replacement was taken due to housing demolition, uninhabited, households change, no response or rejection to this survey, and it was replaced by other one in the neighbor village. The family structure of replacement households was similar to the original household. The replacement percentage was less than 10%. Finally, a total of 3591 participants had finished the face to face interview and blood pressure measurement at their homes with a valid response rate of 98.4%.

### Data collection

Face to face interviews were conducted by trained and experienced staff from local CDC in a private room at participants’ home. Data were collected and managed using a pre-tested questionnaire, which was consisted of socio-demographic characteristics, family history of hypertension, physical activity and FFQ about food intake history both frequency of consumption (daily, weekly, monthly, yearly or never) and quantity of intake of a list of foods and beverages in the past 12 months. There were 26 food groups. Finally, they were merged into 20 groups (rice, wheaten food, coarse cereals, tuber, pork, beef and mutton, animal organ, poultry, aquatic products, vegetables, fruits, eggs, yogurt, milk, beans, soy products, cakes, juice and beverage, oil and salt) due to the similarity of nutrient profiles and the low intake of some food groups.

### Anthropometric measurement

Body weight was measured in light indoor clothing without shoes to the nearest 0.1 kg. Height was measured without heavy clothes and shoes to the nearest 0.1 cm. All measurements were repeated three times during the visit by trained physician using a standard protocol and techniques. Body mass index (BMI) was calculated as weight in kilograms divided by height in meters squared. Underweight, normal weight, overweight and obesity were defined as BMI<18.5, 18.5–23.9, 24.0–27.9, and ≥ 28.0 kg/m^2^, respectively, according to Chinese standard [16].

### Blood pressure measurement

Blood pressure was measured twice at the house of the participants after a rest of 5 minutes by the trained interviewer using standard mercury sphygmomanometer and appropriate-sized cuff according to a standard protocol, and the blood pressure was recorded in average. The hypertension was defined as an average SBP at least 140 mmHg, and/or DBP at least 90 mmHg without any anti-hypertensive medication use [19].

### Definitions of variables

The classification of sex was male and female. Age was divided into four groups: 18–35, 36–45, 46–55, ≥56. The category of ethnicity was classified into four groups: Tibetan, Lisu, Naxi, Han and other. Education level was divided into three groups in terms of years at school: < middle school (< 9 years), middle school (9 years) and > middle school (> 9 years). BMI was classified into four groups according to Chinese standard: underweight (BMI<18.5), normal weight (BMI = 18.5–23.9), overweight (BMI = 24.0–27.9) and obesity (BMI ≥ 28.0). The physical activity level was measured by metabolic equivalents (MET) in minutes per week according to occupation, transportation, housework, and recreational activity, and it was divided into three groups: low (≤599 MET-min/week), moderate (600–2999 MET-min/week), vigorous (≥3000 MET-min/week) [20]. Family hypertension history was defined as diagnosis of hypertension in one parent, and it was categorized as yes, no and unknown.

### Statistical analysis

The data were entered in EpiData 3.1 and analyzed by IBM SPSS 20.0 (IBM SPSS Inc., USA). In all, 3591 subjects were included in the study. Demographic characteristics, BMI, physical activity, and family history of hypertension were defined as categorical variables, and the prevalence of hypertension was calculated in each subgroup. Chi-square test was used to determine significant risk factors for hypertension in Table 1.

**Table 1:** Prevalence of hypertension in Diqing, Yunnan, China (*N* = 3591)

Characteristics	n	Prevalence of hypertension		*p-*value
		n^a^	%	
Sex				0.106
Male	1396	447	32.0	
Female	2195	647	29.5	
Age				0.000
18–35	1049	90	8.6	
36–45	858	186	21.7	
46–55	704	246	34.9	
56-	980	572	58.4	
Ethnicity				0.132
Tibetan	1368	479	35.0	
Lisu	562	112	19.9	
Naxi	810	228	28.1	
Han	551	190	34.5	
Other	300	85	28.3	
Education				0.000
< Middle school	2248	794	35.3	
Middle school	985	203	20.6	
> Middle school	358	97	27.1	
BMI				0.000
Underweight	266	47	17.7	
Normal	2017	467	23.2	
Overweight	937	374	39.9	
Obesity	371	206	55.5	
Physical activity				0.000
Low	2207	753	34.1	
Moderate	995	254	25.5	
Vigorous	389	87	22.4	
Family history of hypertension				0.000
Yes	638	213	33.4	
No	2209	549	24.9	
Unknown	744	332	44.6	

n: the number of participants in each subgroup. n^a^: the number of participants diagnosed as hypertension in each subgroup. Chi-square tests were used to determine if there were any significant differences between hypertension and each subgroup

Dietary patterns were analyzed by factor analysis with principal component based on the 20 food groups from the FFQ. The number of factors retained was determined by eigenvalues (> 1.0), the Scree test and the interpretability of factors. Varimax orthogonal rotation was performed to simplify the factor matrix and facilitate data interpretation by generating nonrelated factors. Rotated factor loadings > 0.25 or < − 0.25 were considered to significantly contribute to a pattern. The highest factor loadings were considered when identifying a name for each of the dietary patterns. The factor analysis generated three major dietary patterns. Pattern-specific scores were calculated and assigned to each participant, and they were divided into quintiles. Socio-demographic characteristics were described across quintiles in each dietary pattern. Chi-square tests were used to determine if there were any significant differences between the groups of demographic characteristics classified in the 1st and 5th quintile for a specific dietary pattern.

Associations between each dietary pattern and hypertension were tested by binary logistic regression with potential confounders adjusted. All statistical tests were two-tailed, and a *p*-value < 0.05 was considered statistically significant.

## Results

### Prevalence of hypertension

The overall prevalence of hypertension aged 18 years old or over among Han and multi-ethnic population in Diqing, Yunnan Province was 30.5% in our study. The prevalence of hypertension both for men and women was 32.0% and 29.5%, respectively. The prevalence of hypertension did not significantly differ across sex and ethnicity. People of the youngest age had the lowest prevalence of hypertension. As age increased, prevalence of hypertension also increased (*p* < 0.01). In addition, the prevalence of hypertension differed significantly over the groups defined by education, BMI, physical activity and family history (Table 1).

### Dietary patterns

Three major types of dietary patterns, namely ‘Grassland healthy’, ‘Tuber and meat’, and ‘fruit and vegetable’ were identified, which totally explained 25.4% of the variance in food intake. The first type was highly relative with yogurt, soy products and eggs. The second type characterized by high intake of tuber, beef and mutton, and pork. The third type was highly correlated with fruits and vegetables. The factors loading for each dietary pattern were presented in Table 2. When demographic characteristics of subjects across quintile categories of each dietary pattern scores were analyzed, we found that participants who were younger and had higher level of education adhered more to the ‘Grassland healthy’ pattern. The male, younger, Tibetan were more likely to involve in the ‘Tuber and meat’ pattern. Participants who were male, younger, Naxi and participants had lower level of education were more likely in the ‘Fruit and vegetable’ pattern, which was presented in Table 3.

**Table 2:** Factor loading of dietary patterns in Diqing, Yunnan, China (*N* = 3591)

Food items	Grassland healthy	Tuber and meat	Fruit and vegetable
Yogurt	**0.76**	0.02	−0.04
Soy products	**0.70**	0.04	0.06
Eggs	**0.65**	−0.10	0.22
Milk	**0.53**	0.13	−0.13
Coarse cereals	0.24	0.08	−0.03
Cakes	0.20	0.13	−0.03
Pork	0.15	**0.60**	0.12
Wheaten food	0.10	**0.35**	0.21
Juice and beverage	0.08	0.09	0.07
Aquatic product	0.06	0.03	0.09
Oil	0.05	0.02	−0.16
Rice	0.04	0.06	0.02
Tuber	0.03	**0.67**	0.13
Beans	0.02	0.02	−0.06
Fruits	0.02	−0.06	**0.74**
Vegetables	0.02	0.16	**0.74**
Animal organs	0.00	0.00	−0.04
Salt	−0.02	0.02	0.20
Beef and mutton	−0.03	**0.67**	−0.12
Poultry	−0.10	−0.01	0.03

In bold are the rotated factors loading > 0.25 or < − 0.25. The three types of pattern explained 11.3%, 7.5% and 6.6% of the variance in dietary intake, respectively

**Table 3:** Socio-demographic characteristics of participants across quintiles categories of dietary pattern scores in Diqing, Yunnan, China (*N* = 3,5 s91)

Demographic characteristics	Grassland healthy		Tuber and meat		Fruit and vegetable	
	Q1	Q5	Q1	Q5	Q1	Q5
	n (%)	n (%)	n (%)	n (%)	n (%)	n (%)
Sex						
Male	265 (37.2)	282 (39.3)	245 (34.2)	363 (50.4)^***^	236 (32.9)	312 (43.8)^***^
Female	448 (62.8)	436 (60.7)	471 (65.8)	357 (49.6)	482 (67.1)	400 (56.2)
Age						
18–35	164 (23.0)	315 (43.9)^***^	213 (29.7)	239 (33.2)^***^	180 (25.1)	207 (29.1)^***^
36–45	186 (26.1)	114 (15.9)	147 (20.5)	178 (24.7)	169 (23.5)	202 (28.4)
46–55	148 (20.8)	124 (17.3)	117 (16.3)	159 (22.1)	137 (18.6)	139 (19.5)
56-	215 (30.2)	165 (23.0)	239 (33.4)	144 (20.0)	232 (32.3)	164 (23.0)
Ethnicity						
Tibetan	238 (33.4)	223 (31.1)	203 (28.4)	332 (46.1)^***^	360 (50.1)	200 (28.1)^***^
Lisu	94 (13.2)	171 (23.8)	114 (15.9)	115 (16.0)	109 (15.2)	84 (11.8)
Naxi	230 (32.3)	128 (17.8)	182 (25.4)	155 (21.5)	135 (18.8)	219 (30.8)
Han	98 (13.7)	142 (19.8)	154 (21.5)	74 (10.3)	71 (9.9)	137 (19.2)
Other	53 (7.4)	54 (7.5)	63 (8.8)	44 (6.1)	43 (6.0)	72 (10.1)
Education						
< Middle school	515 (72.2)	308 (42.9)^***^	462 (64.5)	420 (58.3)	504 (70.2)	434 (61.0)^**^
Middle school	131 (18.4)	313 (43.6)	182 (24.5)	231 (32.1)	150 (20.9)	204 (28.7)
> Middle school	67 (9.4)	97 (13.5)	72 (10.1)	69 (9.6)	64 (8.9)	74 (10.4)

n: number of participants in each quintile. Q1: 1st quintile. Q5: 5th quintile. Chi-square tests were used determine if there were any significant differences between the 1st quintile and 5th quintile of each dietary pattern score regards to participants’ demographic characteristics. ^**^p for trend < 0.01, ^***^p for trend < 0.001

### Dietary patterns and hypertension

The prevalence of hypertension decreased across quintiles of three dietary patterns. As the scores of ‘Grassland healthy’, ‘Tuber and meat’ and ‘Fruit and vegetable’ dietary pattern increased, the prevalence of hypertension decreased from 33.7 to 23.3%, 35.1% to 26.7%, 34.4% to 28.4%, respectively, which was shown in Table 4.

**Table 4:** Prevalence of hypertension across quintiles of dietary patterns in Diqing, Yunnan, China (*N* = 3591)

Dietary pattern	Q1		Q2		Q3		Q4		Q5		*p* for trend
	n	%	n	%	n	%	n	%	n	%	
Grassland healthy	240	33.7	259	36.0	234	32.4	194	27.1	167	23.3	0.000
Tuber and meat	251	35.1	234	32.3	225	31.3	192	27.0	192	26.7	0.000
Fruit and meat	247	34.4	233	32.3	210	29.0	202	28.2	202	28.4	0.003

n: the number of participants detected as hypertension in each quintile

Participants in the 5th quintile of scores for the three dietary patterns were at a lower risk of hypertension. The OR comparing the 1st quintile of scores for the three dietary patterns quintiles to the highest was 0.693 (95% CI: 0.537–0.893), 0.678 (95% CI: 0.530–0.868), 0.759 (95% CI: 0.593–0.970), respectively, after adjusting for sex, ethnicity, education level, BMI, physical activity and family history of hypertension. After further adjustment for age, the association between ‘Grassland healthy’ pattern and hypertension was appreciably attenuated. The negative correlations between the other two dietary patterns and hypertension were disappeared in this study. Table 5 shows the adjusted OR and its 95% CI for hypertension in quintiles of different dietary patterns.

**Table 5:** Adjusted odds ratio for hypertension across quintiles of dietary patterns in Diqing, Yunnan, China (*N* = 3591)

Dietary patterns	Q1	Q5	*p*-value
	Adjusted OR (95% CI)	Adjusted OR (95% CI)	
Grassland healthy			
Model 1	1.000(reference)	0.693 (0.537–0.893)	0.005
Model 2	1.000(reference)	0.703 (0.535–0.924)	0.012
Tuber and meat			
Model 1	1.000(reference)	0.678 (0.530–0.868)	0.002
Model 2	1.000(reference)	0.796 (0.611–1.037)	0.090
Fruit and vegetable			
Model 1	1.000(reference)	0.759 (0.593–0.970)	0.028
Model 2	1.000(reference)	0.842 (0.648–1.094)	0.199

Model 1: adjusted for sex, ethnicity, education level, BMI, physical activity and family history of hypertension

Model 2: model 1 additionally adjusted for age

## Discussion

In the present study, we report the prevalence of hypertension and risk factors of hypertension in univariate analysis which were used for further adjustment. Then we identify three major dietary patterns and examine their associations with hypertension. Thus, we divide the discussions into three parts as follows. Firstly, the overall prevalence of hypertension among Han and multi-ethnic groups aged 18 years and above was 30.5% in Diqing in Yunnan Province, southwest China, whereas the prevalence of hypertension was 21.5% among Chinese residents older than 15 years using a systematic review and meta-analysis between 2002 and 2011 from nine provinces of China [21]. And the disparity of this prevalence in southern Zhejiang Province and northeastern Jiling Province in China is 24.6% [22] and 30.8%, respectively [23]. The prevalence of hypertension in our study is similar with those in northeastern parts of China. Although the differences in methods and study population restrict comparison across studies, the prevalence of hypertension in this study exceeded the results in southern China. Possible explanations for this increase of hypertension prevalence may include the following reasons: first of all, we speculate that the higher prevalence of hypertension is possibly attributed to the environmental factor compared with the low altitude area. Though we did not collect data on the altitude and climate for individual participant, we inquiry the altitude of the habitat for Han, Tibetan, Naxi, Lisu and other ethnic group in Diqing, southwest China which is about 3300 m above sea level. We note that the Tibetan living at the highest altitude had a higher prevalence of hypertension than the ethnic group of Lisu living at the lowest altitude. As a previous study revealed, the prevalence of hypertension increased with altitudes [24]. Secondly, the difference is likely due to difficult access to medical care in this undeveloped area. The majority of rural populations do not regularly see a doctor because of their low income, which implies that a large proportion of hypertensive patients were not early diagnosed and they did not receive any medical treatment and control. As previous studies noted, prevalence of hypertension among ethnic minorities varied between 25% in Hani minority and 64% in Tibetan in Yunnan Province. However, the rates of hypertension treatment and control were lower in Yunnan. Only 19.9% hypertensive patients were treated by diet and/or medication, 11.7% for treatment with medication, and 10.8% for control [25]. These rates of treatment and control were among the lowest in China. A previous study reported the hypertension treatment and control rates was 48.2%, 32.1%, respectively in urban China [26]. Additionally, the prevalence of hypertension we observed in this study was possibly underestimated, because we had excluded the subjects who used anti-hypertension medicine.

Secondly, age, lower education, obesity and family history are related to higher prevalence of hypertension in this study, which is consistent with previous studies [22, 23]. The prevalence of hypertension increases from 8.6 to 58.4% between 18 and 35 years and ≥ 56 years. The oldest group has the highest prevalence of hypertension, compared with the youngest group. The previous studies also reported that the prevalence rates of hypertension was 57.7% (≥60 years) and 53.5% (55–64 years) in southern and northern China, respectively [22, 23]. It appears that age plays a significant role in developing hypertension. Vigorous physical activity is related to lower prevalence of hypertension in our study, and evidence from international studies had further confirmed that physical activity has a favorable effect on blood pressure reduction [27]. However, the prevalence of hypertension did not differ across the various minorities in the present study. Inconsistent with this, a previous multi-ethnic population survey reported that some minorities were associated with a higher prevalence rate of hypertension compared to Han population [28]. Previous studies usually selected different places and various dietary habits for comparison. We assume that the discrepancy of prevalence of hypertension was possibly reduced by the adaptation to local environment and diet among multi-ethnic population living in this area.

Thirdly, we identified three major dietary patterns named the ‘Grassland healthy’, ‘Tuber and meat’ and ‘Fruit and vegetable’ pattern among Han and multi-ethnic population in Diqing, Yunnan Province, southwest China. These three dietary patterns were negatively associated with hypertension after adjustment of sex, ethnicity, education, BMI, physical activity and family history of hypertension. When age was additionally adjusted, the ‘Grassland healthy’ pattern was inversely correlated with hypertension, while there were no associations between the other two dietary patterns and hypertension. The ‘Grassland healthy’ pattern characterized by high intake of yogurt, soy products and eggs, which was not similar with traditional diets in south China, characterized by high intake of fruit, pork, poultry, rice, vegetables, aquatic products [29, 30]. This could be interpreted by ethnic and regional differences. Tibetan and other minorities preferred milk and dairy products in their diet culture, and yogurt was highly available in this pastoral area enriched by fertile grassland. Yogurt is a fermented dairy product. A previous study shows that regular yogurt intake could reduce the risk of cardiovascular disease among hypertensive adults [31]. Egg protein, milk protein, soy protein and pea protein can reduce systolic blood pressure and diastolic blood pressure among overweight men and women with pre-hypertension or untreated stage I hypertension [32]. The prevalence of hypertension decreased from 33.7 to 23.3% as the scores of the ‘Grassland healthy’ pattern increased in our study. This dietary pattern had been identified as a protective factor for developing hypertension after adjustment of confounders. Our finding is consistent with studies in different counties. A study from Taiwan reported that egg and dairy food pattern had a significant lower risk of hypertension [33]. Another study from Pakistan reported that the yogurt pattern was less likely to be associated with hypertension [34]. The ‘Tuber and meat’ dietary pattern we identified in this study was characterized by high intake of tuber, beef and mutton, and pork. Although this pattern adopters consumed red meat which was widely recognized as a risk factor for hypertension [35], this dietary pattern was appeared to be a protective factor for hypertension in the present study. Accordingly, we further adjusted for age in the multi-variable logistic regression. The correlation between the ‘Tuber and meat’ pattern and the prevalence of hypertension did not exist, which indicated that the association could be more attributed to participants’ age. Compared with the meat pattern which was defined as high intake of chickens, beef, lamb, duck, goose in a previous study [36], the ‘Tuber and meat’ pattern in the present study was loaded heavily on tuber. The tuber rich in dietary fiber was reported to be inversely associated with obesity and positively associated with underweight [37]. The dietary fiber has the antihypertensive effects as well as the prevention of a number of cardiovascular diseases [38, 39]. The dietary pattern named ‘Fruit and vegetable’ was correlated with high intake of fruit and vegetable in our study. As we know, fruits, vegetables, and low-fat dairy products which were characterized by Dietary Approaches to Stop Hypertension (DASH) pattern had been regarded as one of the most well-known dietary strategies for antihypertensive effect [40, 41]. The negative association between this pattern and hypertension was appeared to be found, but the association was more likely to be explained by participants’ age. When age was controlled, this inverse relationship disappeared. Even the ‘Tuber and meat’ dietary pattern and ‘Fruit and vegetable’ dietary pattern were not related to the lower prevalence of hypertension. It is also suggested that a healthy diet is an important strategy to prevent and control hypertension for us.

Additionally, the three dietary patterns also captured the socio-demographic characteristics of the study participants. Participants who were younger and had higher level of education adhered more to the ‘Grassland healthy’ pattern. High socioeconomic status assessed by higher education, purchasing power, or urban location was generally significantly associated with a healthy diet, which had been also reported in a systematic review [42]. The male, younger, Tibetan were more likely to involve in the ‘Tuber and meat’ pattern. As previously reported that the Tibetan had a preference for a special local diet of tsampa, butter tea, beef, and mutton [43]. Participants who were male, younger, Naxi and had lower level of education were more frequently involved in the ‘Fruit and vegetable’ pattern. Our finding revealed the individuality in minority’s diets in China.

Nevertheless, the present study had several limitations. First, this study was cross-sectional in nature, thus the causal relationship between dietary patterns and hypertension could not be determined. Second, the semi-quantitative FFQ enabled us to capture more reliable information on participants’ dietary patterns, but the recall bias could not be eliminated. Thirdly, the study participants were recruited from Diqing, southwest China, making a regional and ethnic representation. Therefore, it might not be generalized to other places and population.

## Conclusions

In conclusion, our results indicate that ‘Grassland healthy’ dietary pattern is associated with lower risk of hypertension, providing an additional support for dietary intervention strategies to reduce the risk of hypertension among multi-ethnic populations. Considering the high prevalence of hypertension reported in this study, larger prospective studies are required to confirm our findings, and a scientific rationale for hypertension prevention is needed in Yunnan, especially in the economically depressed area.

## Additional file

Additional file 1: Informed consent. (DOC 28 kb)

## Abbreviations

95% CI: 95% confidence interval
BMI: Body mass index
CDC: Centre for Disease Control and Prevention
DASH: Dietary Approaches to Stop Hypertension
DBP: Diastolic blood pressure
FFQ: Food frequency questionnaires
MET: Metabolic equivalents
OR: Odds ratio
SBP: Systolic blood pressure
