# Supplementary material for: Association between dietary patterns and hypertension among Han and multi-ethnic population in southwest China
Source: BMC Public Health. 2018 Sep 10;18:1106. doi: 10.1186/s12889-018-6003-7 (PMC6131804; doi:10.1186/s12889-018-6003-7)
Supplement: Supplementary file 1 — Informed consent. (DOC 28 kb) [file 12889_2018_6003_MOESM1_ESM.doc]

Informed Consent

Dear friend：

You will be invited to take part in a study. This informed consent gives you some information to help you decide whether to participate or not. Please read it carefully. If you have any questions, please ask the researchers who are responsible for this.

The study is used for the purpose of investigating your habitual diet and the association between diet and health. It is supported by public health projects of the Centre for Disease Control and Prevention in Diqing, Yunnan, southwest China. If you agree to participate in this study, we will ask you some questions, provide physical examination, and measure your blood pressure. Please note, all of these are free. For you, all information will be confidential.

We acknowledge your participation and support. If you agree to take part in this study, please mark as “√” in the box below and sign your name.

I am voluntary to participate in the study.

Your signature:

Date:
